# 16.3 METHYLOMIC CHANGES OF OXIDATIVE STRESS REGULATION, AXON GUIDANCE AND INFLAMMATORY PATHWAYS DURING CONVERSION TO PSYCHOSIS

**DOI:** 10.1093/schbul/sby014.062

**Published:** 2018-04-01

**Authors:** Oussama Kebir, Boris Chaumette, Marie-Odile Krebs

**Affiliations:** 1 CH Sainte Anne, Paris; 2 INSERM U894 - Ste Anne Hospital; 3 INSERM U894, Paris Descartes University, Sainte-Anne Hospital, Service Hospitalo-Universitaire

## Abstract

**Background:**

Epigenetics is hypothesized to mediate the interplay between genes and environment leading to the onset of psychosis

**Methods:**

We performed a longitudinal prospective study of genomic DNA methylation during psychotic transition in help-seeking young individuals referred to a specialized outpatient unit for early detection of psychosis and enrolled in a 1-year follow-up (n=39). We used Infinium HumanMethylation450 BeadChip array after bisulfite conversion and analyzed longitudinal variations in methylation at 411947 cytosine–phosphate–guanine (CpG) sites.

**Results:**

We observed that conversion to psychosis was not associated with a global change in methylation and there was no individual CpG significantly associated with psychotic transition. By contrast, we found that conversion to psychosis was associated with specific methylation changes in genes involved in axon guidance, as well as genes of the IL-17 pathway and the glutathione-S-transferase family.

**Discussion:**

Alterations in oxidative stress regulation, axon guidance and in inflammatory pathways could represent multiple theaters for the disruption in homeostasis that accompanies the emergence of full-blown psychosis.

